# Artemisia argyi essential oil modulates gut microbiota to influence serum metabolism in rabbits: effects on growth, meat quality, and organ index

**DOI:** 10.1128/spectrum.00223-26

**Published:** 2026-07-22

**Authors:** Huyang Bao, Ziru Zhang, Xiuzhu Sun, Zhanjun Ren, Shuhui Wang

**Affiliations:** 1 College of Animal Science and Technology, Northwest A&F University 12469, Yangling, China; 2 College of Grassland Agriculture, Northwest A&F University 12469, Yangling, China; Chengdu University, Chengdu, Sichuan, China

**Keywords:** hycole rabbit, *Artemisia argyi *essential oil, cecal microbiota, serum metabolism.

## Abstract

This study aims to evaluate the effects of *Artemisia argyi* essential oil (AAEO) on the growth, meat quality, carcass performance, organ index, intestinal microbiota, and serum metabolome of *Hycole* rabbits. A total of 96 Hycole rabbits, aged 35 days, were randomly assigned to four groups, with six replicates per group and four rabbits per replicate. The rabbits were fed a basal diet (without AAEO) or diets supplemented with 100, 200, or 300 mg/kg of AAEO for 35 days. Supplementation with AAEO at 100 mg/kg increased the average daily feed intake (ADFI) and the organ index of the sacculus rotundus, whereas the addition of 300 mg/kg AAEO to the diet resulted in a reduction in ADFI compared to the control group (*P <* 0.05). AAEO intervention enhanced the colonization of *Cloacibacillus* and *[Eubacterium]_ruminantium_group* in the cecum of rabbits (*P <* 0.05). A total of 120 differential metabolites were identified in the serum, which were primarily enriched in glycerophospholipid metabolism and linoleic acid metabolism pathways. Integrated analysis revealed consistency between the cecal microbiota and serum metabolites. In summary, dietary supplementation with AAEO at 100 mg/kg optimizes gut microbial composition, alters serum lipid absorption and metabolic processes, and contributes to improved health in rabbits.

**IMPORTANCE:**

This study provides a scientific basis for using traditional Chinese medicinal herbs as feed additives in rabbit production. The findings hold significant value for enhancing rabbit meat quality, decreasing antibiotic dependency, and promoting the production of healthier meat products for human consumption.

## INTRODUCTION

With the improvement of economic levels and health consciousness among consumers, the market share of nutritious meat has been steadily expanding (1). Rabbit production has gradually evolved into a highly specialized industry in both China and Europe, owing to its inherent advantages such as adaptability to diverse climatic zones, short life cycles, and strong sustainability (2). Nowadays, nutritional intervention through the addition of green feed additives has become a practical approach to enhance meat quality traits in livestock and poultry (3, 4). *Artemisia argyi* Levl. et Vant. (*A. argyi*), an important member of the Asteraceae family, possesses significant medicinal potential and practical value. As an extract derived from *A. argyi* leaves, AAEO primarily contains chemical components such as cis-butene, globulol, and 1,8-cineole (5). Following oral administration in rats, AAEO exhibits the highest gastrointestinal exposure and demonstrates multiple pharmacological activities both *in vivo* and *in vitro*, including antibacterial, antitumor, antioxidant, and immunomodulatory effects (6–8).

The diversity of gut microbiota is closely associated with host health (9–11). Intestinal microorganisms play crucial roles in maintaining intestinal barrier function, enhancing nutrient absorption, and dynamically regulating host metabolism and immune responses (12, 13). Recent studies have demonstrated that gut microbes ferment carbohydrates or dietary fibers to produce short-chain fatty acids (SCFAs), which directly modulate lipid metabolism (14, 15). Current research has revealed the beneficial effects of AAEO in preventing *Salmonella pullorum* infection in chicks by modulating the gut microbiota (16). Additionally, intervention with AAEO in mice on a high-fat diet downregulated the abundance of *Alistipes* and *Rikenella*, thereby influencing lipid metabolic absorption processes (17, 18).

However, there is limited evidence regarding whether dietary AAEO can modulate gut microbiota and serum metabolism in rabbits. This study aimed to evaluate the effects of AAEO supplementation in rabbit diets on meat quality, carcass traits, and growth performance, while demonstrating its potential as a feed additive for enhancing rabbit health.

## MATERIALS AND METHODS

### Animal and treatments

AAEO was purchased from Hubei Daming Ancient Ai Technology Co., Ltd. (Jichun, China). The chemical composition of the AAEO used in this study has been previously characterized in detail (19). A total of 96 35-day-old Hycole rabbits with a 1:1 male-to-female ratio were randomly selected from the population and divided into four treatment groups. AAEO was added to the basal diet at concentrations of 0 mg/kg (C), 100 mg/kg (L), 200 mg/kg (M), and 300 mg/kg (H). Each group contained six replicates with four rabbits per replicate. Based on the nutritional requirements recommended by the Chinese Standard for Rabbit Feed (NY/T 4049-2021), a basal diet was formulated (Table 1). The content of each fatty acid (%) in the complete diet was calculated as the sum of the weighted contributions from each ingredient using the following formula: Diet FA (%) = Σ_*i*_ [Ingredient Proportion (%) × Ingredient FA Content (%)], where “FA” represents a specific fatty acid, and “*i*” represents an individual ingredient. The specific data were provided by China Feed Data (https://chinafeeddata.org.cn/admin/Login/index.html). The fatty acid data for sunflower meal were obtained from Razzaghi et al. (20), while the crude fat data for corn germ meal are from Qi et al. (21).

**TABLE 1:** Ingredients and nutrient compositions of the experimental control diet (%, as-fed basis)

Item	Content
Ingredients	
Corn	6.0
Flour	3.0
Wheat bran	22
Alfalfa	3.0
Soybean oil	1.3
Soybean meal	7.0
Sunflower meal	9.0
Corn germ meal	23.7
Rice husk powder	12.0
Limestone meal	1.3
Premix	3.0
Total	100.0
Nutrient composition	
AME^*a*^, MJ/kg	2,416.10
Crude protein	16.66
Crude ash	8.11
Crude fiber	15.87
Crude fat	3.03
Calcium	1.25
Total phosphorus	0.53
Lysine	0.99
Methionine	0.68
Cysteine	0.29
Threonine	0.51
Lignin	5.16
Fatty acid profile (% total fatty acids)	
C12:0	0.14
C14:0	0.17
C16:0	13.17
C16:1*n*−7	0.20
C18:0	2.49
C18:1*n*−9	21.93
C18:2*n*−6	55.84
C18:3*n*−3	5.91
C20:0	0.02
C20:1*n*−9	0.02
C22:0	0.07
C24:0	0.02
SFA	16.09
MUFA	22.15
PUFA	61.76

^*a*^ AME, apparent metabolizable energy.

Feed three times a day at 9:00 am, 6:00 pm, and 9:00 pm, with free access to food and water. During the experiment, the indoor temperature is controlled at 22°C ± 2°C, and humidity is maintained at 55%–60%, with natural ventilation and lighting. The animal pens were cleaned regularly. The health status of the experimental rabbits is observed and recorded daily.

### Growth performances

Body weights were measured on days 0, 7, 14, 21, 28, and 35 at 8:00 AM after a 12-h fast. Daily feed intake was recorded by weighing provided and residual feed. Average daily gain (ADG), average daily feed intake (ADFI), and feed conversion ratio (FCR) were calculated.

### Sample collection

After the feeding trial, 5 mL of blood was collected from the ear vein into non-additive vacutainer tubes (Genenode, Wuhan, China). The blood was left to stand at room temperature for 30 min and then centrifuged at 17,800 × *g* for 10 min to collect the serum, which is then stored in a −80°C freezer. All rabbits were euthanized using excessive isoflurane (RWD, Shenzhen, China). Samples of cecal content were collected and transferred immediately into liquid nitrogen and stored in a −80°C freezer. The longissimus dorsi muscle samples (two pieces per rabbit) were collected to evaluate the meat quality traits. Weight of heart, liver, spleen, lung, kidneys, vermiform appendix, and sacculus rotundus was measured to evaluate the performance of carcass traits and organ indices (22).

### Carcass performance

Upon completing the trial, a single animal was chosen at random from every replicate unit. Six rabbits were included in each group, and 5 mL of blood was collected from the ear vein into non-additive vacutainer tubes (Genenode, Wuhan, China). Blood samples were collected and processed as described in the “Sample collection” section. On the final experimental day, a total of 24 rabbits were transported 269 km to the laboratory at the College of Grassland Agriculture, Northwest A&F University for slaughter. All rabbits were allowed to rest with free access to water for 12 h prior to slaughter, after which their body weight was recorded as the pre-slaughter live weight. All rabbits were euthanized using excessive isoflurane (RWD, Shenzhen, China). Following Wang et al. (3), processed rabbits (containing head, renal, hepatic, and thoracic tissues) were stored at 4°C for 24 h. The weight was recorded as the commercial slaughter weight (CCW). Utilizing the recorded weight, the commercial dressing percentage (CDP) was derived. All rabbits provided samples of the *Longissimus thoracis et lumborum* (*LTL*) muscle for subsequent examination. The *LTL* muscle samples were collected from the same anatomical location on the left side of each carcass. The samples were divided into two portions: one was used to measure pH and color at 45 min post-slaughter, followed by additional pH measurement after 24 h storage at 4°C; the remaining samples were snap-frozen in liquid nitrogen and then maintained at −80°C.

### Meat quality

The pH was measured with a pH-star device (automatic temperature compensation function; MATTHAUS, Germany) at 45 min and 24 h post slaughter at different sites, with the average of three readings taken. The pH probe was previously calibrated with pH 4.0 and 7.0 standard buffers. All the muscle samples were allowed to bloom for 45 min at 4°C. Meat color was assessed with a MiniScan EZ 4500 colorimeter (HunterLab, USA) equipped with an 8 mm aperture, 0% UV filter, and a D-65/10° illuminant/observer. Measurements were taken at various spots on the *LTL*, and the average of three readings was recorded for L* (lightness), a* (redness), and b* (yellowness). For cooking loss, the meat samples were cut into cubes (1 cm × 1 cm × 2 cm), weighed, placed in sealed polyethylene bags, and cooked in 80°C water bath for 30 min. Samples from all treatment groups were randomized in the order of cooking to ensure that any potential, unmeasured drift in the equipment over time would not systematically bias one treatment group over another. After cooking, the samples were cooled to room temperature, surface water was removed with a paper towel, and weighed. The cooking loss percentage was calculated using the following formula (23):


$$\text{Cooking loss}(\%)=\frac{\text{weight before cooking}-\text{weight after cooking}}{\text{weight before cooking}}\times 100$$

For drip loss, weigh 20 g of the longest back muscle sample, remove peripheral fat, and cut it into cubes (2 cm × 2 cm × 4 cm); the weighed muscle samples were hung in sealed polyethylene bags at 4°C and weighed again after 24 h to calculate the drip loss. Calculate the percentage of drip loss using the following formula:


$$\text{Drop loss}(\%)=\frac{\text{weight before storage}-\text{weight after storage}}{\text{weight before storage}}\times 100$$

The cooked samples were used for shear force analysis. The samples were cut into pieces with fibers parallel to the long axis and measured using a C-LM3B Texture Analyzer (Tenovo, China). Each value represents the average of three measurements.

### Organ index

Record the weight of the rabbit and the weight of its heart, liver, spleen, lung, kidneys, vermiform appendix, and sacculus rotundus in detail and calculate the organ index using the following formula:


$$\text{Organ index}(\%)=\left(\frac{\text{organ weight (g)}}{\text{weight before storage (kg)}}\right)\times 100$$

### Cecal microbiota analysis

The cecum contents of 8 rabbits (Group C and Group L with 4 rabbits in each group) were randomly selected for 16S rRNA analysis. After slaughter, cecal contents from six randomly selected rabbits per group were gathered into 5 mL cryogenic vials and homogenized. The V3-V4 region of the bacterial 16S rRNA gene was amplified using primers 338F (5ʹ-CCTAYGGGRBGCASCAG-3ʹ) and 806R 5ʹ-GGACTACNNGGGTATCTAAT-3ʹ) with TransStart FastPfu DNA Polymerase (Transgen Biotech, China). Purified amplicons were sequenced on an Illumina NovaSeq platform (Novogene, China). Raw reads were processed using QIIME2 (Version QIIME2-2), and ASVs (amplicon sequence variants) were clustered with DADA2 (24). Alpha diversity (Chao1, Shannon, Simpson) and beta diversity (Non-Metric Multi-Dimensional Scaling) were analyzed (25).

### Serum metabolomics analysis

Serum metabolomics of 8 (Group C and Group L with four rabbits in each group) randomly selected rabbits were studied. A 100 μL aliquot of each sample was processed with 400 μL of methanol-acetonitrile (V:V = 2:1, containing a mixture of internal standards at 4 μg/mL) for pretreatment. Analysis was performed using a LC-MS system consisting of a Waters ACQUITY UPLC I-Class Plus coupled with a Thermo QE HF high-resolution mass spectrometer. Chromatographic separation was achieved on an ACQUITY UPLC HSS T3 column (100 mm × 2.1 mm, 1.8 μm) using 0.1% formic acid (solvent A) and acetonitrile (solvent B) as mobile phases. The elution gradient is detailed in Table S1. Mass spectrometry conditions are provided in Table S2. Mass spectra were acquired in positive/negative ion modes. Metabolites were identified using HMDB (http://www.hmdb.ca/), Metlin (https://metlin.scripps.edu/), and LuMet-Animal 3.0 databases. All identified differential metabolites reported in this study were annotated at confidence Level 2.

Data preprocessing (peak alignment, normalization) and OPLS-DA analysis were performed with Progenesis QI (Nonlinear Dynamics, Newcastle, UK). Perform a statistical test between two treatment groups to screen based on *P* value, VIP, and Fold Change (FC). If the *P* value is less than 0.05 and the VIP value is greater than 1, the metabolite is considered to have a significant difference.

Differential metabolite pathway analysis was performed using MetaboAnalyst (https://www.metaboanalyst.ca). Metabolic pathways were visualized utilizing bioinformatics (https://www.bioinformatics.com.cn/).

### Combined microbiome-metabolome analysis

For Procrustes analysis, the cecal microbial and serum metabolomic data sets were first preprocessed separately. Microbial ASV counts were rarefied and converted to relative abundances (%). Metabolite peak areas were normalized by total ion intensity. Both data sets were then log-transformed, and each variable was autoscaled. Principal component analysis (PCA) was performed on each preprocessed data set, and the sample scores on the first few principal components were used for Procrustes analysis on the Oebiotech cloud platform (https://cloud.oebiotech.com). Correlation analysis between the top 50 most abundant genera and the top 50 differential metabolites was performed using Spearman’s rank correlation, with clustering based on correlation coefficients. To account for multiple comparisons, *P* alues were adjusted using the Benjamini-Hochberg false discovery rate (FDR) method.

### Statistical analysis

IBM SPSS Statistics (version 27.0, IBM Corp, Armonk, New York, USA) was used to analyze the data on growth performance, slaughter traits, meat quality traits, and organ indices through one-way ANOVA. Duncan’s method was employed to compare differences among various groups, and linear and quadratic regression analyses were conducted to assess the dose-response effects of AAEO in the diet. Differences in microbial abundance at the genus level were analyzed using the Mann-Whitney *U* test. Significant differences are denoted by different letters or one asterisk (*) for *P* < 0.05, whereas two asterisks (**) indicate *P* < 0.01, signifying a greater level of statistical significance.

## RESULTS

### Growth properties

The results presented in Table 2 indicate a significant difference in the ADFI between the control group and L treatment (*P* < 0.05), while the H treatment significantly reduced ADFI (*P* < 0.05). During the experiment, with the increase of AAEO level, the reaction showed quadratic patterns (*P* < 0.05).

**TABLE 2:** The impact of AAEO on the growth performance of rabbits^*a*^

Item^*b*^	Groups^*c*^				SEM	*P* value		
	C	L	M	H		ANOVA	Linear	Quadratic
IBW, kg	1.00 ±0.01	1.00 ± 0.01	1.02 ±0.01	1.01 ±0.01	0.004	0.436	0.191	0.378
FBW, kg	2.31 ±0.03	2.34 ±0.03	2.28 ±0.03	2.24 ±0.04	0.015	0.186	0.088	0.072
ADG, g/d	40.21± 0.88	40.95± 0.70	39.41± 0.62	37.69± 1.34	0.466	0.080	0.151	0.038
ADFI, g/d	155.06± 1.8^b^	158.81 ± 0.9^a^	153.69± 1.3^b^	150.23 ± 1.3^c^	1.380	0.003	0.120	0.002
F:G	3.88 ±0.09	3.90 ± 0.07	3.92 ±0.06	4.02 ± 0.13	0.043	0.702	0.843	0.506

^*a*^ Values in the same row with no common letter superscripts mean significant difference (*P* < 0.05).

^*b*^ IBW, initial body weight; FBW, final body weight; ADG, average daily gain; ADFI, average daily feed intake; F:G, gain to feed ratio; SEM, Standard error of the mean.

^*c*^ C, basic diet without supplementation of AAEO; L, supplemented with 100 mg/kg AAEO; M, supplemented with 200 mg/kg AAEO; H, supplemented with 300 mg/kg AAEO. Data are presented as mean ± SEM (*n* = 6 per group).

### Carcass performance and meat quality

As shown in Tables 3 and 4, no significant differences were observed in slaughter performance or meat quality between rabbits fed varying doses of AAEO and the control group (*P* > 0.05).

**TABLE 3:** The effect of AAEO on the slaughtering performance of rabbits^*a*^

Item	Groups^*b*^				SEM	*P* value		
	C	L	M	H		ANOVA	Linear	Quadratic
Pre-slaughter live weight, kg	2.08±0.02	2.05±0.02	2.11±0.02	2.15±0.05	0.016	0.130	0.064	0.079
CCW, kg	1.09±0.02	1.06±0.01	1.11±0.02	1.13±0.04	0.012	0.177	0.095	0.150
CDP, %	0.52±0.01	0.52±0.00	0.53±0.01	0.53±0.01	0.002	0.504	0.472	0.757

^*a*^ Values in the same row with no common letter superscripts mean significant difference (*P *< 0.05). Values in the same row with no common uppercase letter superscripts mean significant difference. (*P *< 0.001).

^*b*^ C, basic diet without supplementation of AAEO; L, supplemented with 100 mg/kg AAEO; M, supplemented with 200 mg/kg AAEO; H, supplemented with 300 mg/kg AAEO. Data are presented as mean ± SEM (*n *= 6 per group).

**TABLE 4:** The impact of AAEO on the quality of domestic rabbit meat^*a*^

Item^*b*^	Groups^*c*^				SEM	*P* value		
	C	L	M	H		ANOVA	Linear	Quadratic
L*	31.11±0.30	32.16±0.47	31.42±0.20	31.94±0.40	0.189	0.452	0.880	0.657
a*	3.94±0.14	4.74±0.73	3.37±0.16	3.65±0.24	0.230	0.103	0.271	0.452
b*	4.94±0.45	5.18±0.24	4.52±0.35	4.16±0.23	0.184	0.266	0.507	0.600
pH_45min_	6.26±0.08	6.40±0.13	6.32±0.11	6.58±0.06	0.052	0.063	0.061	0.177
pH_24h_	5.88±0.04	5.82±0.05	5.86±0.04	5.88±0.05	0.021	0.777	0.912	0.641
Drip loss, %	3.00±0.28	3.10±0.31	3.12±0.26	3.40±0.11	0.125	0.758	0.062	0.188
Cooking loss, %	32.36±1.26	32.81±1.16	31.95±0.25	32.66±1.24	0.546	0.965	0.968	0.991
Shear force, *N*	39.07±6.32	29.16±4.12	32.03±4.69	34.34±5.19	2.515	0.585	0.145	0.009

^*a*^ Values in the same row with no common letter superscripts mean significant difference (*P *< 0.05). Values in the same row with no common uppercase letter superscripts mean significant difference. (*P *< 0.001).

^*b*^ L*, lightness; a*, redness; b*, yellowness.

^*c*^ C, basic diet without supplementation of AAEO; L, supplemented with 100 mg/kg AAEO; M, supplemented with 200 mg/kg AAEO; H, supplemented with 300 mg/kg AAEO. Data are presented as mean ± SEM (*n *= 6 per group).

### Organ index

The results in Table 5 indicate that different doses of AAEO significantly influence the organ index of sacculus rotundus in rabbits. The organ index of sacculus rotundus increased at a supplementation level of 100 mg/kg (*P* < 0.05).

**TABLE 5:** The impact of AAEO on the organ index of rabbits^*a*^

Item	Groups^*b*^				SEM	*P* value		
	C	L	M	H		ANOVA	Linear	Quadratic
Heart, %	0.27 ±0.01	0.28 ±0.01	0.27±0.01	0.27 ±0.01	0.005	0.747	0.738	0.859
Liver, %	2.50 ±0.09	2.52 ±0.02	2.60±0.07	2.53 ±0.03	0.029	0.670	0.636	0.736
Spleen, %	0.06 ±0.00	0.06 ±0.01	0.05±0.00	0.07 ±0.00	0.002	0.063	0.608	0.066
Lung, %	0.70 ±0.07	0.55 ±0.04	0.64±0.06	0.59 ±0.04	0.028	0.279	0.704	0.775
Kidney, %	0.31 ±0.02	0.29 ±0.01	0.29±0.00	0.29 ±0.01	0.006	0.490	0.264	0.290
Vermiform appendix, %	0.35 ±0.01	0.37 ±0.02	0.36±0.01	0.37 ±0.02	0.007	0.744	0.324	0.610
Sacculus rotundus, %	0.11 ±0.01^ab^	0.12 ± 0.00^a^	0.10±0.00^b^	0.11 ±0.01^ab^	0.003	0.007	0.814	0.968

^*a*^ Values in the same row with no common letter superscripts mean significant difference (*P *< 0.05). Values in the same row with no common uppercase letter superscripts mean significant difference. (*P* < 0.001).

^*b*^ C, basic diet without supplementation of AAEO; L, supplemented with 100 mg/kg AAEO; M, supplemented with 200 mg/kg AAEO; H, supplemented with 300 mg/kg AAEO. Data are presented as mean ± SEM (*n *= 6 per group).

### Alterations in the ecological structure of cecal microbiota in in rabbits fed with AAEO

Given that varying levels of AAEO supplementation did not influence slaughter performance or meat quality, the significant increase in sacculus rotundus weight and ADFI at 100 mg/kg indicated its potential for immune support. This dose was, thus, considered the optimal dose for further investigation, leading to a focused comparison of the cecal microbiota and serum metabolomes between the C and L treatment groups.

**Fig 1:**
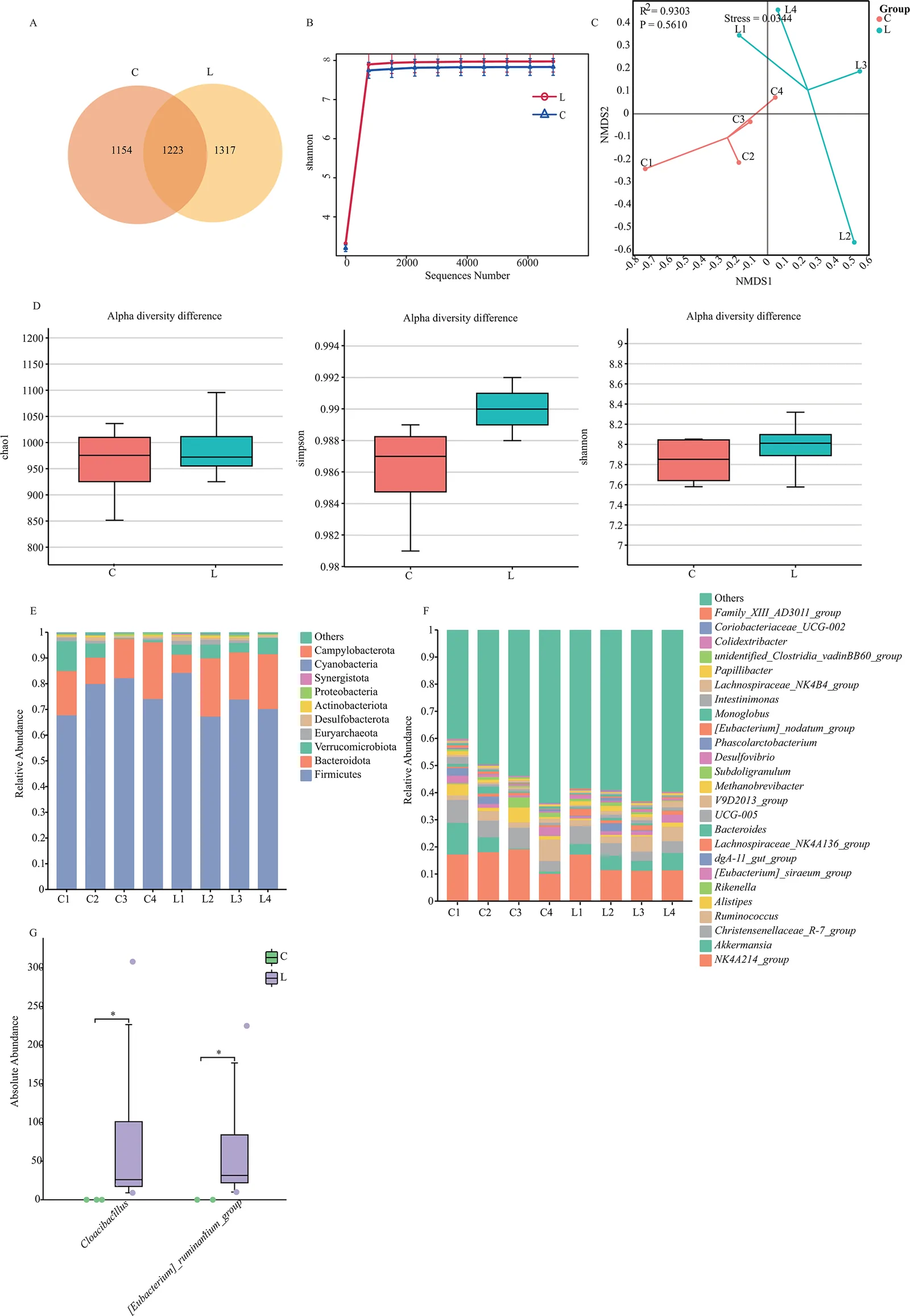
Cecal microbiome analysis between the C and L treatments: (**A**) Venn diagram of ASV sequences in the C and L treatments. (**B**) Shannon curves of microbial diversity indices. Different colors represent different samples. (**C**) NMDS plot. Each point represents an independent sample. (**D**) Box plot of microbial alpha diversity. Differences in Chao1, Shannon, and Simpson indices in the cecum of rabbits from the C and L treatments. (**E**) Stacked bar plot of species abundance at the phylum level across samples. The horizontal axis represents samples, and different colors indicate different species. (**F**) Stacked bar plot of species abundance at the genus level across samples. (**G**) Box plot of differentially abundant species at the genus level between the C and L treatments. Different colors denote different groups. C, basic diet without supplementation of AAEO; L, supplemented with 100 mg/kg AAEO (*n* = 4).

Since diet is a critical factor influencing the gut microbiota, and AAEO intervention altered the organ indices of gut-associated lymphoid tissues, this study investigated the cecal microbial composition in rabbits. There was a total of 1,223 ASVs sequences in groups C and L, 1,154 sequences specific to group C, and 1,317 sequences specific to group L after clustering and noise reduction with 100% similarity using DADA2 (Fig. 1A). This high-resolution approach ensures the reliability of subsequent data analyses.

Based on the microbial diversity index Shannon curves constructed using rabbit cecum content samples with different sequencing depths (Fig. 1B), the sequence curves of the different groups of samples are relatively flat, indicating that the sequencing depths are sufficient to accurately reflect the information of most microorganisms present in the rabbit cecum content, which meets the test requirements. The alpha diversity of the cecum microbiota, including Simpson, Chao1, and Shannon diversity, as shown in Fig. 1D, was not affected by the addition of wormwood essential oil to the diet on any of the alpha indexes of diversity of the cecum flora (*P* > 0.05). NMDS was employed to investigate the variation in beta diversity (Fig. 1C). The stress value of 0.034 indicates the results are representative. The community structure of L treatment showed no significant difference compared to other groups (*P* > 0.05). Fig. 1E shows the relative abundance percentages of the top 15 sequences detected at the phylum level and the top 25 sequences detected at the genus level for each treatment. Firmicutes, Bacteroidota, and Verrucomicrobiota were the dominant bacteria in cecum of rabbit, with a total abundance of more than 95%. At the genus level, analysis using Mann-Whitney *U* method revealed that, compared to the C treatment, the abundances of *Cloacibacillus* and *[Eubacterium]_ruminantium_group* showed significant differences.

### Metabolic profiles in serum of rabbits of the C and L groups

**Fig 2:**
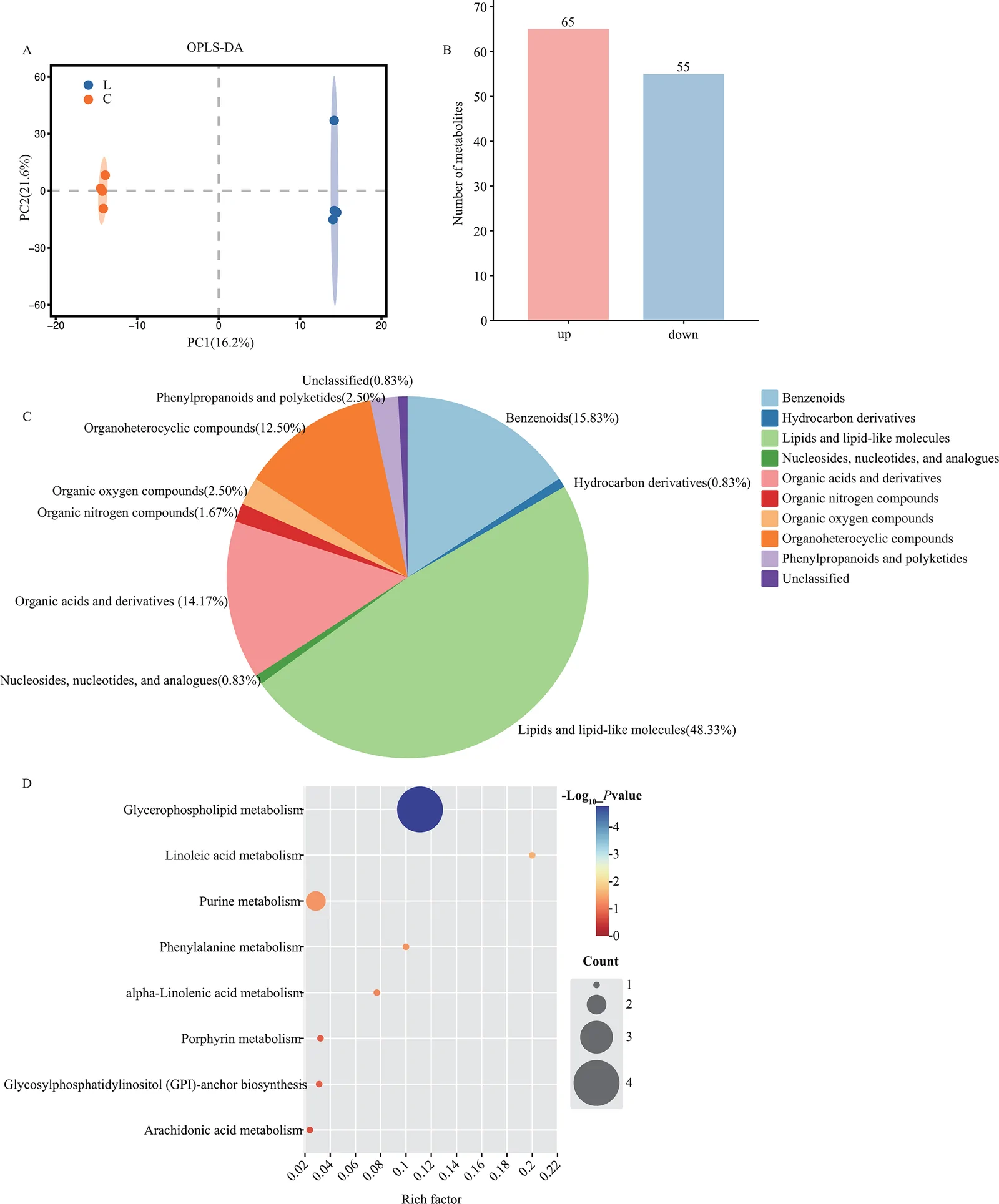
Metabolomic profiling of serum from group C and group L: (**A**) OPLS-DA score plot. PC1 and PC2 indicate the first and second principal components, respectively. Each dot represents an individual sample, ellipses denote 95% confidence intervals, and colors distinguish the two treatment groups. (**B**) DMs bar chart. Up- and downregulated metabolites are indicated by distinct colors. (**C**) DMs class pie chart. Each color represents a different metabolite category. (**D**) KEGG enrichment analysis of DMs between treatment groups C and L. The *x*-axis indicates the rich factor for each pathway, the *y*-axis lists the pathway names, dot size reflects the number of mapped metabolites. Color is related to *P* value, the redder the color, the greater the *P* value, and the bluer the color, the smaller the *P* value. C, basic diet without supplementation of AAEO; L, supplemented with 100 mg/kg AAEO (*n* = 4).

**Fig 3:**
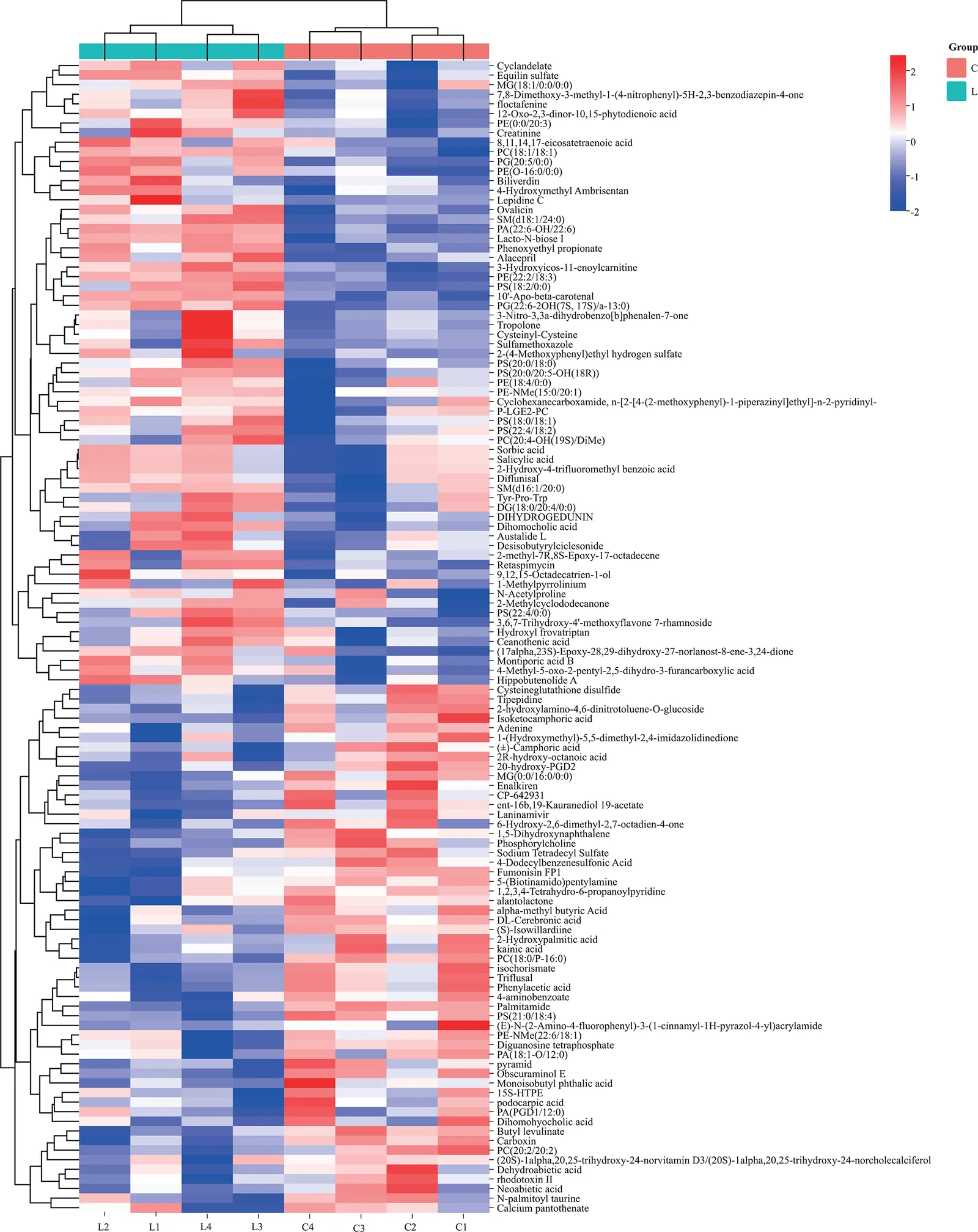
Hierarchical clustering heatmap of DMs: Columns correspond to samples, rows to metabolites; red indicates high abundance, blue low abundance. C, basic diet without supplementation of AAEO; L, supplemented with 100 mg/kg AAEO (*n* = 4).

To further investigate the impact of gut microbiota composition on host metabolism, we profiled the serum metabolome of AAEO fed rabbits using LC-MS. Metabolomic differences among groups were assessed by OPLS-DA (Fig. 2A). A significant separation between groups was observed in the score plot of the OPLS-DA model, indicating that AAEO intervention significantly altered the serum metabolite composition (*R*^2^*X* = 0.33, *R*^2^*Y* = 0.998, *Q*^2^ = 0.265, *P*＜0.05) (Fig. 2A). Database search was performed using The Human Metabolome Database (HMDB), Lipidmaps (v2.3), METLIN, and LuMet-Animal3.0, leading to the detection of 2,566 metabolites. DMs were identified under the criteria of *P* < 0.05 and VIP > 1, yielding 120 DMs, among which 65 were upregulated and 55 were downregulated (Fig. 2B). These DMs primarily consisted of lipids and lipid-like molecules, benzenoids, and organic acids and derivatives (Fig. 2C). A heatmap illustrating the levels of DMs across samples is shown in Fig. 3. Upregulated metabolites mainly included PE (22:2/18:3), PG (22:6-2OH(7S, 17S)/a-13:0), Lacto-N-biose I, Alacepril, and PS (20:0/20:5-OH(18R)). Downregulated metabolites included PS (21:0/18:4), phosphorylcholine, palmitamide, carboxin, and obscuraminol E, among others.

DMs were imported into MetaboAnalyst 3.5 for KEGG enrichment analysis to further explore the potential mechanisms by which AAEO improves rabbit production performance (Fig. 2D). The results revealed that DMs were predominantly enriched in the following metabolic pathways: glycerophospholipid metabolism, linoleic acid metabolism, purine metabolism, phenylalanine metabolism, alpha-linolenic acid metabolism, porphyrin metabolism, glycosylphosphatidylinositol (GPI)-anchor biosynthesis, and arachidonic acid metabolism.

### The relationship between cecal microbiota and serum metabolites

**Fig 4:**
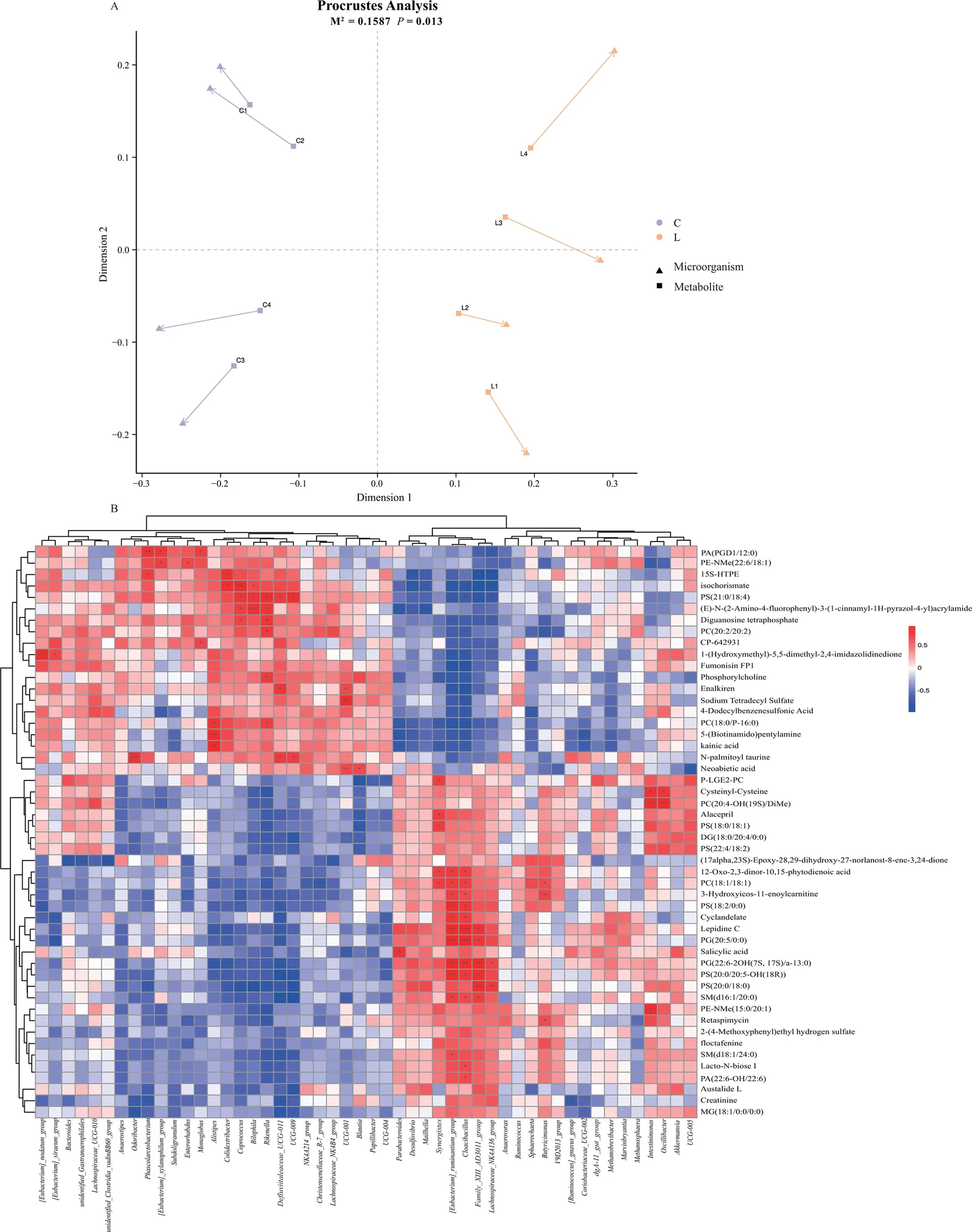
Combined analysis of microbiome and metabolome in rabbit. (**A**) Procrustes analysis between microbial and DMs. Colors represent different treatment groups, with triangles denoting metabolites and squares representing microbial. (**B**) Clustered heatmap of Spearman correlation analysis between microorganisms and the top 50 DMs. Rows represent metabolites, and columns represent microorganisms. Red indicates positive correlations, and blue indicates negative correlations, with color intensity corresponding to the strength of the correlation. C, basic diet without supplementation of AAEO; L, supplemented with 100 mg/kg AAEO. Numerical representation as mean ± SEM (*n* = 4). *P < 0.05, **P < 0.01, ***P < 0.001.

We used PCA to perform dimensionality reduction sorting on the genus level microbiome and metabolome, followed by Procrustes analysis (Fig. 4A). The analysis shows *M*^2^ = 0.1587, *P* = 0.013, indicating consistency between changes in microbial abundance at the genus level and changes in serum metabolites.

Pearson correlation analysis was performed between the top 50 cecal microbes at the genus level and serum metabolites. The results revealed that *[Eubacterium]_ruminantium_group* and *Cloacibacillus* showed significant positive correlations with PC (18:1/18:1), PS (20:0/20:5-OH(18R)), SM (d16:1/20:0), PG (20:5/0:0), PS (18:2/0:0), PG (22:6-2OH(7S, 17S)/a-13:0), 3-Hydroxyicos-11-enoylcarnitine, Cyclandelate, Lepidine C, and 12-Oxo-2,3-dinor-10,15-phytodienoic acid. Conversely, significant negative correlations were observed with 4-Dodecylbenzenesulfonic Acid, Phosphorylcholine, Fumonisin FP1, PC (18:0/P-16:0), 5-(Biotinamido)pentylamine, PS (21:0/18:4), 1-(Hydroxymethyl)−5,5-dimethyl-2,4-imidazolidinedione, kainic acid, isochorismate, and Enalkiren (Fig. 4B).

## DISCUSSION

Growth performance represents one of the most phenotypic indicators. Current studies have indicated that natural plant extracts can exert positive effects on animal growth (26, 27). However, consistent with the findings of Liu et al., (28) dietary supplementation with AAEO did not significantly influence the growth traits of rabbits. Compared to the control group, the ADFI increased significantly at the supplementation level of 100 mg/kg, while further elevation in AAEO dosage led to a reduction in ADFI. This outcome is supported by the research of Niu (29) who suggested that unpleasant flavors associated with certain natural plant constituents may affect feed palatability. Specifically, they reported that although low-level EA supplementation significantly improved the ADG in weaned piglets and numerically increased ADFI, higher EA levels resulted in declines in both ADFI and ADG.

Organ indices serve as critical parameters reflecting the impact of diet on animal development and physiological function. Well-developed immune organs are closely correlated with immune responsiveness and growth performance (30, 31). The present study revealed that AAEO supplementation altered the organ index of sacculus rotundus. A substantial body of literature highlights the role of rabbit gut-associated lymphoid tissue (GALT), particularly in transporting luminal antigens and functioning as intestinal immune sensors (32–34). Beyaz et al. (35) observed unique structural characteristics in the macrophage populations within the sacculus rotundus, noting the presence of multiple apoptotic bodies—a feature distinct from macrophages in other mammalian organs—indicating potent phagocytic activity. Furthermore, evidence suggests that the gut microbiota composition is functionally linked to the immune performance of the sacculus rotundus (36, 37). These findings imply that AAEO supplementation may enhance immune function in rabbits.

Regarding meat quality, while most traits were unaffected, shear force showed a notable numerical difference between the control and AAEO-treated groups. However, this difference did not reach statistical significance (*P* = 0.145). A post-hoc power analysis was performed. The analysis yielded a statistical power of 0.51, indicating that the study had only 51% power to detect a true difference of this magnitude. Therefore, the non-significant *P* value should be interpreted with caution, and the observed improvement in tenderness may warrant further investigation with a larger sample size.

As dietary supplementation with AAEO at varying levels did not alter slaughter performance or meat quality traits in rabbits, the significant increase in sacculus rotundus weight observed at 100 mg/kg suggests a potential enhancement of immune function. Furthermore, the ADFI was significantly higher at this supplementation level compared to other treatments, whereas the H treatment exhibited a reduction in ADFI relative to the control. Based on these findings, we propose that a dietary supplementation level of 100 mg/kg AAEO may represent the optimal dosage. Consequently, subsequent analyses focused on comparing the cecal microbiota and serum metabolome profiles between the C and L treatment groups.

The gut microbiota represents the largest and most complex microbial ecosystem in animals, playing a crucial role in host health through functions such as nutrient absorption, metabolism, and immunity (38). Existing evidence indicates that AAEO supplementation does not affect the gut microbial composition at the phylum level in mice fed a high-fat diet, which is consistent with the findings of this study that no significant changes were observed between the C and L treatments (17). Research by Hu et al. (39) on microbial composition across different tissues in Belgium gray rabbits revealed that the abundance of Firmicutes was significantly higher in intestinal tissues compared to non-intestinal tissues. In this study, Firmicutes showed the highest relative abundance in the cecal microbiota, accounting for over 73% in all groups. Bacteria within this phylum are primarily involved in processes such as SCFA production and cholesterol metabolism. At the genus level, a non-parametric Mann–Whitney *U* test was applied to compare the abundance of *[Eubacterium]_ruminantium_group*, a member of the Firmicutes phylum, across groups (40). The results revealed a significant increase in its abundance in the L treatment group. *Eubacterium* species are key fiber-degrading bacteria that contribute to gastrointestinal homeostasis by decomposing carbohydrates to produce SCFAs like butyrate and propionate, as well as unsaturated fatty acids such as palmitoleic acid (41, 42). These SCFAs supply energy to the host, promote the proliferation and differentiation of intestinal epithelial cells, and help maintain the mechanical barrier function of the intestinal mucosa (43, 44). In ruminants, the abundance of *[Eubacterium]_ruminantium_group* is positively correlated with muscle fatty acid deposition and feed efficiency (45, 46). AAEO supplementation was associated with an increased relative abundance of *Cloacibacillus*. Previous studies have demonstrated that *Cloacibacillus* can metabolize glycated proteins such as N-ε-carboxymethyllysine in the gut (47). However, the functional role of Cloacibacillus in the gut is context-dependent. Therefore, the positive correlation between Cloacibacillus abundance and serum metabolites observed in the present study should be interpreted as a correlational finding, and the functional contribution of this genus to host metabolism in rabbits remains to be determined. Supplementation with AAEO in BALB/c mice significantly upregulates the abundance of *Akkermansia* and *Bifidobacterium* (48). Similarly, in this study, AAEO increased the colonization of *Akkermansia* in rabbit intestines though the change was not statistically significant—a discrepancy that may be attributed to species differences. Nevertheless, both genera were more abundant in AAEO-fed rabbits, indicating that dietary AAEO supplementation enriches SCFA-producing bacteria and promotes a balanced and beneficial gut microbial community.

Serum metabolomics provides valuable insights into the correlations between host phenotypes and serum metabolites, particularly those associated with lipid metabolism and protein synthesis. Animal studies have confirmed that dietary supplementation with AAEO improves lipid metabolism by modulating adipose tissue weight and serum lipid levels, including HDL and LDL (17). LC–MS analysis identified 120 DMs between the C and L treatment groups, which were primarily classified into categories such as lipids and lipid-like molecules, benzenoids, and organic acids and derivatives. DMs including PE (22:2/18:3), PG (22:6-2OH(7S,17S)/a-13:0), Lacto-N-biose I, Alacepril, PS (20:0/20:5-OH (18R)), PS (21:0/18:4), phosphorylcholine, palmitamide, carboxin, and obscuraminol E. Among these, the changes in glycerophospholipids such as PG (22:6-2OH(7S,17S)/a-13:0), PS (20:0/20:5-OH (18R)), and PS (21:0/18:4) were consistent with the enrichment of the glycerophospholipid metabolism pathway in KEGG analysis, suggesting that AAEO may have globally regulated the lipid metabolism network. PG (22:6-2OH(7S,17S)/a-13:0), PS (20:0/20:5-OH(18R)), and palmitamide have been reported to possess anti-inflammatory and immunomodulatory functions (49, 50). Specifically, PG (22:6-2OH(7S,17S)/a-13:0) has been shown to inhibit the NF-κB pathway by downregulating ERK phosphorylation and nuclear translocation of p65 and p50, thereby suppressing the expression and production of inflammatory mediators such as IL-6 and CCL5 (51). The changes in these lipids signaling molecules may be potentially associated with the enhancement of immune function in rabbits after AAEO intervention.

Notably, the upregulated differential metabolite Lacto-N-biose I is a known prebiotic closely associated with changes in the abundance of gut bifidobacteria (52). In addition, studies have shown that Lacto-N-biose I can competitively inhibit pathogen binding to the intestinal epithelium and modify antigen-presenting cells independently of Th1-type immunity, inducing the production of antigen-specific IL-4 (53). However, no significant changes in the abundance of *Bifidobacterium* species were observed in this study, suggesting that the role of Lacto-N-biose I may be mediated through other mechanisms that are not yet clear.

In summary, AAEO may exert positive effects on rabbit health by regulating serum metabolite levels, thereby influencing lipid metabolism, intestinal health, and inflammatory status.

Furthermore, KEGG enrichment analysis of these DMs highlighted key pathways including glycerophospholipid metabolism, linoleic acid metabolism, and purine metabolism. These findings align with those reported by Wang et al., (18) who demonstrated that AAEO supplementation in mice significantly influences linoleic acid and glycerophospholipid metabolic pathways. Glycerophospholipids are essential structural components of cell membranes and exhibit considerable molecular diversity (54–56). *De novo* synthesized glycerophospholipids undergo acyl transfer through the action of phospholipases, generating fatty acid derivatives, lysophospholipids, and other lipid mediators that play crucial roles in animal biology and physiological functions (57, 58). Zhang et al. (59) demonstrated that elevated glycerophospholipid levels are associated with epithelial repair and anti-inflammatory signaling. The linoleic acid metabolism pathway contributes to the production of SCFAs, which help alleviate inflammation. This suggests that AAEO supplementation primarily modulates lipid absorption and metabolism through these pathways. Procrustes analysis indicated a consistent variation between microbial composition at the genus level and serum metabolite abundance. Spearman correlation analysis revealed positive correlations between *Cloacibacillus* and *[Eubacterium]_ruminantium_group* and upregulated glycerophospholipid metabolites including PC (18:1/18:1), PS (20:0/20:5-OH(18R)), SM (d16:1/20:0), PG (20:5/0:0), PS (18:2/0:0), and PG (22:6-2OH(7S, 17S)/a-13:0). Conversely, negative correlations were observed with downregulated metabolites such as 4-Dodecylbenzenesulfonic Acid, PC (20:2/20:2), Fumonisin FP1, and PC (18:0/P-16:0). Animal studies have established a strong link between SCFAs and glycerophospholipid metabolism (60–62). Acetate provides a substrate for phosphoglycerol synthesis, while butyrate promotes glucagon-like peptide-1 (GLP-1) secretion in L-cells via G protein-coupled receptors (GPR41), influencing adipocyte lipid deposition and metabolic status through the AMPK signaling pathway (63, 64). Based on these correlative findings, we hypothesize that AAEO supplementation may enhance SCFA levels by increasing the abundance of specific colonizing bacteria. It is plausible that these SCFAs could then be released into the bloodstream and may subsequently influence glycerophospholipid and linoleic acid metabolism in rabbits. However, direct measurement of SCFAs and further intervention studies are required to validate this proposed mechanism.

This study proposes the use of AAEO as a sustainable strategy to modulate metabolic pathways by reshaping the gut microbiota composition through microbial metabolic mechanisms. Specifically, dietary AAEO supplementation was associated with an increased abundance of *Cloacibacillus* and *[Eubacterium]_ruminantium_group*, which were correlated with metabolites involved in glycerophospholipid metabolism and linoleic acid metabolism pathways. However, this research has several limitations that should be acknowledged. One limitation pertains to pre-slaughter logistics. Rabbits were transported approximately 269 km by road prior to slaughter. Transport stress is known to induce physiological changes, including cortisol elevation and altered metabolic profiles, which may directly affect serum metabolites and meat quality traits such as pH decline and drip loss. Specifically, stress-induced activation of the hypothalamic-pituitary-adrenal (HPA) axis leads to glucocorticoid release, which can promote lipolysis and increase circulating free fatty acids, thereby altering glycerophospholipid metabolism. In addition, catecholamines released during acute stress can shift lipid metabolism toward β-oxidation and modify phospholipid remodeling pathways (65). Consequently, the serum levels of lipid mediators such as PG (22:6-2OH (7S,17S)/a-13:0) and PS (20:0/20:5-OH (18R)) reported in this study may reflect, in part, a combination of AAEO effects and stress responses. Although all treatment groups were subjected to identical transport and fasting conditions, and group-wise comparisons are, therefore, still meaningful, the absolute values reported should be interpreted with caution. Future studies should consider on-site slaughter facilities to minimize transport stress and better isolate the effects of dietary interventions. Another limitation concerns the scope of multi-omics analysis. 16S rRNA sequencing and serum metabolomics were performed only for the C and L groups. The H group, which showed significantly reduced ADFI, was not analyzed at these levels due to sample depletion. Consequently, the mechanistic basis for this adverse effect at the higher dose remains unknown. The conclusions regarding gut microbiota and serum metabolism, therefore, apply only to the 100 mg/kg dose under the conditions tested.

In addition, cecal SCFAs were not directly quantified. Although the enrichment of SCFA-producing genera such as *[Eubacterium]_ruminantium_group* is consistent with increased SCFA production, the causal link between microbial shifts, SCFA levels, and glycerophospholipid metabolism remains hypothetical. Targeted quantification of SCFAs is necessary to test this hypothesis in future studies.

### Conclusion

This study shows that dietary supplementation with 100 mg/kg AAEO, without affecting slaughter performance or meat quality, significantly increased ADFI and the organ index of the sacculus rotundus, suggesting a potential enhancement of immune function in rabbits. Furthermore, this intervention was associated with positive changes in cecal microbiota composition and function. Specifically, AAEO supplementation at 100 mg/kg was associated with an increased abundance of *Cloacibacillus* and *[Eubacterium]_ruminantium_group*. Correlation analysis further suggested a potential link between these microbial changes and the modulation of serum glycerophospholipid and linoleic acid metabolism pathways. We propose that this might occur through microbiota-mediated production of SCFAs, a hypothesis that requires direct experimental validation in future studies. Notably, the higher dose reduced ADFI without additional benefits, indicating a non-linear dose response; therefore, the present findings are limited to the 100 mg/kg dose and should not be generalized to higher doses. These results provide new ideas for healthy rabbit breeding and reducing antibiotic use, while highlighting the importance of dose optimization.

## Data Availability

The 16S rRNA sequencing data have been provided with the accession number PRJNA1457301 in the Materials and Methods section and can be searched in the NCBI database. The serum metabolomics data have been provided with the accession number PRJCA048741 in the same section and can be accessed via the NGDC Genome Sequence Archive. The raw metabolomics data files have been deposited in the OMIX database under accession number OMIX017940.

## SUPPLEMENTAL MATERIAL

Supplemental tables spectrum.00223-26-s0001.docx Tables S1 and S2.

ASM does not own the copyrights to Supplemental Material that may be linked to, or accessed through, an article. The authors have granted ASM a non-exclusive, world-wide license to publish the Supplemental Material files. Please contact the corresponding author directly for reuse.
