# Supplementary material for: Artemisia argyi essential oil modulates gut microbiota to influence serum metabolism in rabbits: effects on growth, meat quality, and organ index
Source: Microbiol Spectr. 2026 Jul 22;14(9):e00223-26. doi: 10.1128/spectrum.00223-26 (PMC13537472; doi:10.1128/spectrum.00223-26)
Supplement: Supplementary file 1 [file spectrum.00223-26-s0001.docx]

**SUPPLEMENTAL MATERIAL**

Supplementary Table S1. Elution gradient

| Time | A %^1^ | B %^1^ |
| --- | --- | --- |
| 0.0 | 95.0 | 5.0 |
| 2.0 | 95.0 | 5.0 |
| 4.0 | 70.0 | 30.0 |
| 8.0 | 50.0 | 50.0 |
| 10.0 | 20.0 | 80.0 |
| 14.0 | 0.0 | 100.0 |
| 15.0 | 0.0 | 100.0 |
| 15.1 | 95.0 | 5.0 |
| 16.0 | 95.0 | 5.0 |

^1^ solvent A: water containing 0.1 % formic acid; solvent B: acetonitrile.

Supplementary Table S2. Mass spectrometry parameters

| Parameter | Cation | Anion |
| --- | --- | --- |
| Spray Voltage, V | 3800 | -3200 |
| Capillary Temperature, ℃ | 320 | 320 |
| Aux gas heater temperature, ℃ | 350 | 350 |
| Sheath Gas Flow Rate, Arb | 35 | 35 |
| Aux gas flow rate, Arb | 8 | 8 |
| S-lens RF level | 50 | 50 |
| Mass range, m/z | 70-1050 | 70-1050 |
| Full ms resolution | 60000 | 60000 |
| MS/MS resolution | 15000 | 15000 |
| NCE/stepped NCE^a^ | 10, 20, 40 | 10, 20, 40 |

^a^NCE=Normalized Collision Energy
